# Preoperative Neutrophil Lymphocyte Ratio in Prediction of Adnexal Mass Torsion

**DOI:** 10.1155/2023/3585189

**Published:** 2023-02-25

**Authors:** Asmita Ghimire, Sailaja Ghimire, Asmita Shrestha, Samriddha Raj Pant, Nilam Subedi, Padam Raj Pant

**Affiliations:** ^1^Grande International Hospital, Kathmandu, Nepal; ^2^Bhim District Hospital, Bhairahawa, Nepal; ^3^Tribhuwan University Teaching Hospital, Kathmandu, Nepal

## Abstract

**Aims:**

Adnexal torsion commonly affects reproductive age group females. Prompt diagnosis and early management help in the preservation of fertility. However, its diagnosis is challenging. Preoperative diagnosis can be suspected in only 23–66% of the cases and half of the patients operated for adnexal torsion have different diagnosis. This article thus aims to identify the diagnostic value of preoperative neutrophil lymphocyte ratio in adnexal torsion in comparison with other untwisted unruptured ovarian cysts.

**Methods:**

This was a retrospective study conducted in the duration of five years from 1st January 2016 to 1st January 2020. The data about demographic parameters, hematological parameters, operative approach, operative technique, and histopathological reports were derived from an electronic database and documented on proforma. SPSS was used for statistical analysis. Logistic regression analysis and influence of each factor on preoperative diagnosis of Adnexal torsion was evaluated.

**Results:**

A total of 125 patients were included in the article (adnexal torsion group *n* = 25, untwisted unruptured ovarian cyst group *n* = 100). There was no statistically significant difference in comparison to age, parity, and abortion between both groups. Most patients had undergone laparoscopic surgery which was based on surgeon's skill and preference. Nineteen (78%) patients in the adnexal torsion group underwent oophorectomy while infarcted ovary was seen in only 4 cases. Among the blood parameters, only neutrophil-lymphocyte ratio (NLR) >3 was found to be statistically significant under logistic regression analysis. Most common adnexal pathology to undergo torsion was serous cyst.

**Conclusion:**

Preoperative neutrophil-lymphocyte ratio can be a predictive marker for diagnosis of adnexal torsion and can differentiate it from untwisted unruptured ovarian cysts.

## 1. Introduction

Adnexal torsion is the twisting of the vascular pedicle of an ovary, an ovary with cyst, a fallopian tube, or a paratubal cyst. It results in ischemia of the structure distal to twisting with an acute onset of pain [1]. It accounts for 2.7% to 7.4% gynecological emergencies [2]. The most common age group affected is reproductive age group [3]. Early diagnosis and management of adnexal torsion helps in preservation of fertility; however, its diagnosis is challenging. With detailed history, clinical examination and pelvic ultrasound preoperative diagnosis can be suspected in only 23–66% of the cases and half of the patients operated for adnexal torsion have different diagnosis [4].

In adnexal torsion, ischemia followed by reperfusion causes accumulation of activated neutrophils. This in turn releases reactive oxygen species (ROS) which leads to an increase in inflammatory parameters such as white blood cell (WBC) count, neutrophils, and neutrophil-lymphocyte ratio (NLR) [5]. Adnexal torsion is associated with high inflammatory burden [6]. Elevated NLR is a characteristic of inflammatory conditions shown in various studies. For thyroid disorders, NLR of the subjects with increased uptake in thyroid uptake scintigraphy was significantly higher than those with normal uptake [7]. In the irritable bowel syndrome (IBS) group, NLR > 2.2 was found to be predictive of disease than normal population [8]. Patients with NLR ≥ 2.4 were 20.5 times more likely to have COVID-19 compared to patients whose NLR was ≤2.4 [9]. Similarly, the median NLRs of patients with inflammatory bowel disease presenting with abscesses were significantly higher than in patients without the disease. Moreover, higher ratio suggested acute indication for surgery [10]. For those with uncontrolled diabetes, median NLR of the type 2 DM group >2.4 was found to be significant [11]. Also, patients who had atrial fibrillation with the history of diabetes had higher NLR ratio than patients with atrial fibrillation without diabetes [12]. Thyroiditis patients when assessed also had high NLR of more than 2.1 compared to normal population [13]. These findings of various inflammatory conditions over the decades have showed the importance of elevated NLR in supporting the diagnosis. Same mechanism could be correlated with adnexal torsion. Along with clinical and radiological findings, if hematological parameters can provide additive benefit in the diagnosis of adnexal torsion, then we can be more accurate and plan the management accordingly. However, there is a dearth of published literature exploring the predictive value of neutrophil/lymphocyte ratio in the preoperative diagnosis of adnexal/ovarian torsion.

This article, therefore, aimed to identify if the diagnostic value of neutrophil/lymphocyte ratio in adnexal torsion in comparison with other untwisted unruptured ovarian cyst.

## 2. Methods

This was a retrospective study conducted in Grande International Hospital, Kathmandu, Nepal, after obtaining approval from the institutional review committee of the same institute (approval no. 12/2021). Data of all the gynecological surgeries done over a period of five years from January 1^st^ 2016 to January 1^st^ 2020 were retrieved from the record section/department/database of the institute. All the patients who had undergone surgery for adnexal torsion and ovarian cyst confirmed postoperatively were selected. A total of 213 patients were found who had undergone surgery for adnexal torsion and ovarian cyst in that duration. Patients with a suspicion of malignancy [14], ruptured ovarian cyst [15], tubal ovarian abscess [10], comorbidities such as atherosclerotic heart disease, diabetes mellitus, heart disease (30), and recent surgical procedures [13] were excluded from the study because these may have an effect on blood count parameters. Excluding all these cases, a total of 125 cases were included in the study.

Two groups were formed (adnexal torsion (AT, *n* = 25) and untwisted unruptured ovarian cyst (UOC, *n* = 100)) and investigated. All the data about the demographic parameters (age, parity, and abortion), hematological parameters (WBC count and differential counts), operative approach and technique, and the histopathological reports were obtained from the electronic database and documented in a preformed proforma. SPSS was used for data entry and statistical analysis. Normality of data was assessed by Shapiro–Wilk test. In the study, descriptive and categorical data were evaluated as number (*n*) and percentage (%), and continuous data were studied as interquartile range and medians. Logistic regression analysis and the influence of each factor on the preoperative diagnosis of AT were evaluated. Receiver operating curve (ROC) analysis was used to evaluate the diagnostic value of neutrophil-lymphocyte ratio in predicting AT. Area under the curve (AUC) was used to determine sensitivity and specificity of each marker. *P* value <0.05 was considered as statistically significant. Most important factor in determining adnexal torsion was identified as odds ratio. In the present study, the cut-off value for ROC curve analysis was 3, while it was determined as 8.8 K/L for WBC [16].

## 3. Results

There were a total of 125 patients included in the study (adnexal torsion *n* = 25 and untwisted unruptured ovarian cyst *n* = 100).

### 3.1. Demographic Parameters

The median age group of the patient in study group was 30 years. However, in the AT group, it was 32 years and in UOC group 30 years. Most of the cases were nulliparous in both the groups (44% in AT and 45% in UOC). Most cases had no history of abortion in both groups (Table 1). No statistically significant difference was seen in both groups in comparison to age, parity, and abortion (Table 2).

### 3.2. Blood Parameters

**Figure 1:**
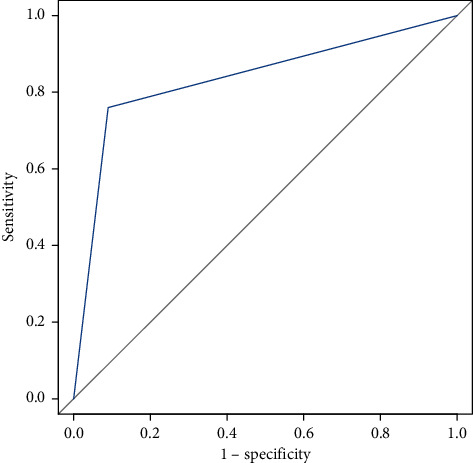
Receiver operating characteristic (ROC) curve of NLR for discriminating patients with an adnexal torsion from ovarian cyst (area under curve (AUC): 0.835 (0.745–0.925)).

The median WBC count in the study group was 8070 cells/mm^3^. In the AT group, it was 9950 cells/mm^3^, and in the UOC group, it was 7475 cells/mm^3^. Neutrophil and lymphocyte levels were 74.40% and 21.20%, respectively, in the AT group and 56.70% and 31.15%, respectively, in UOC group. NLR was 3.47 in AT group and 1.82 in UOC group (Table 3). Though the median value of WBC, neutrophil, and lymphocyte levels showed difference in both groups, it was not statistically significant. However, statistical significant level was seen in the neutrophil-lymphocyte ratio (NLR) between both groups (Table 2). Sensitivity and specificity of NLR in the diagnosis of AT in comparison to UOC were 76% and 91%, respectively (Table 4). Area under the curve was 0.835 (0.745–0.925) (Figure 1). The odds ratio of NLR ratio being ≥3 is 26.345 (95% CI 2.630–263.877) in AT diagnosis compared to UOC diagnosis (Table 2).

### 3.3. Operative Technique

Twenty cases (*n* = 20, 80%) of the AT group and 87 cases of the UOC group (*n* = 87, 87%) underwent laparoscopic surgery. The rest underwent laparotomy. Difference in the operative technique was due to surgeon's preference. In the AT group, 19 cases (76%) had undergone oophorectomy and six cases (24%) cystectomy. However, in the UOC group, 72 cases (72%) underwent cystectomy and 28 cases (28%) underwent oophorectomy (Table 5).

### 3.4. Histopathological Findings

Comparing the histopathological findings, most cases in both groups showed serous tumor followed by mature cystic teratoma. Infarcted ovary was present in only four cases of AT group, revealing that not all cases of ovarian torsion needed oophorectomy (Table 5).

## 4. Discussion

Adnexal torsion, a gynecological emergency, when inaccurately diagnosed could lead to damage of ovaries ultimately affecting the fertility. Complete blood count is the informative proinflammatory marker which is a cheap test and usually performed before every surgery. However, its importance in adnexal torsion has not been studied in detail. Current evidence shows that these blood parameters are useful in diagnosing and prognosticating in case of ischemic diseases such as acute coronary syndrome, acute ischemic stroke, and acute mesenteric ischemia [14, 17, 18]. Normal Doppler flow can be observed in up to 60% of adnexal torsion cases confusing the diagnosis [19].

Out of the total 125 participants, median age group in the AT group was found to be 30 years supporting the fact that adnexal torsion occurs mostly in the reproductive age group [3]. However, there was no significant difference in comparison to UOC group where median age group was 32 years. This is in contrast to the study conducted by Soysal and Baki [20] where the median age group in UOC group was 24 years (19–31). The difference could be because of different inclusion and exclusion criteria. Regarding the parity and abortion, there was no statistical difference between the AT and UOC groups. This was similar to a study conducted by Ercan et al. [16].

White blood cell count in AT group was not statistically significant in comparison to UOC group. This finding is dissimilar to studies conducted by Lee et al., Ercan et al, and Soysal and Baki [5, 16, 20]. In the present article, unadjusted odds ratio (UOR) of neutrophil count and lymphocyte count showed significant difference in both studied groups but adjusted odds ratio (AOR) was not statistically significant. This is also in contrast to other studies [5, 16, 20].This could be because the logistic regression model was used to evaluate the data where many confounders were excluded. However, confounders could be a hindering factor in their study. A study by Tas et al. [15] had used the similar statistical test which supported our findings. Discrepancies between the different study groups could be because of the methodological difference, different hematological analyzers, variation in inclusion and exclusion criteria, and interval between diagnosis and blood parameter assessment.

Comparing the NLR between two groups, there was a statistically significant difference. NLR > 3 was found to be predictive of AT group with a moderate sensitivity (76%) and high specificity (91%) with AUC value (0.835) of good significance. If NLR ratio is ≥3, there are 26 times high possibility of adnexal mass being an adnexal torsion. USG and color Doppler have sensitivity of 22%–66%, which is found to be lesser than that of NLR supported by various studies including the present study [5].

NLR has been a popular inflammatory marker and is important these days. NLR is superior to WBC in the estimation of undesired outcomes within the inflammatory process and surgical conditions [21]. Different studies used the cut-off value of NLR > 3 as a marker for adnexal torsion [5, 16, 20]. The primary pathophysiology in ovarian torsion is ischemia. Following ischemia, reperfusion occurs which may result in the accumulation of the activated neutrophils. Activated neutrophils then release reactive oxygen species [5].

In the torsion of adnexa, ischemia damage to adnexal tissue occurs which produces reactive oxygen species and nitrogen species. This leads to leukocyte activation, resulting in further damage and cell death similar to mechanisms which occur in ischemia of the brain and kidney [22].

Operative technique was as per surgeon's preference. Surgeons in our center preferred laparoscopy. Regarding the surgical procedure, most cases underwent cystectomy. In the AT group, 19 cases (76%) underwent oophorectomy, however, infarcted ovary was seen in only 4 cases, suggesting the fact that not all cases need oophorectomy. These findings were similar to a study conducted by Lee et al. [5]. Depending upon the intraoperative findings and viability of ovarian tissue, conservative surgery can be helpful. Analyzing the HPE reports of the AT group, the commonest finding was mature cystic teatime followed by serous and mucinous tumor, respectively. This was contrary to the study done by Vijayalakshmi et al. where mucinous was seen commonly followed by serous and mature cystic teatime [23]. Mature cystic teatime was found to have undergone more torsion in other studies [24]. The differences could be because of lower number of cases in all the studies.

Thus, the strength of the present article is that it included cases of chronic infection and endometriosis cases which could have affected the level of blood parameters. On logistic regression analysis, AOR showed statistically significant NLR for adnexal torsion. The sensitivity and specificity of NLR showed fairly good supportive results. This supported the fact that in emergency settings for proper diagnosis, NLR can be helpful. Moreover, it is much more helpful in low resource settings where facilities of ultrasonography and color Doppler are unavailable and diagnosis is solely based on clinical and hematological parameters.

The limiting factor of the study is its retrospective nature and small sample size; single center data and symptoms were not included. Moreover, the time interval between symptoms, presentation, blood parameters assessment, and surgery could not be traced. It has been found that the time duration between symptoms and assessment of blood parameters has an effect on the blood parameters. Greater the duration, more the tissue damage and higher the level of blood parameters [5].

Preoperative neutrophil-lymphocyte ratio (NLR) can be a predictive marker for diagnosis of adnexal torsion and can differentiate adnexal torsion from ovarian cysts.

## Figures and Tables

**Table 1:** Demographic parameters of the patients.

Characteristics	Adnexal torsion, *n* (%)	Ovarian cyst, *n* (%)	Total, *n* (%)
Age range			
≤20	5 (20%)	13 (13%)	18 (14.4%)
21–30	7 (28%)	38 (38%)	45 (36%)
31–40	10 (40%)	28 (28%)	38 (30.4%)
41–50	2 (8%)	19 (19%)	21 (16.8%)
51–60	0	1 (1%)	1 (0.8%)
≥61	1 (4%)	1 (1%)	2 (1.6%)
Parity			
0	11 (44%)	45 (45%)	56 (44.8%)
1	4 (16%)	21 (21%)	25 (20%)
2	9 (36%)	29 (29%)	38 (30.4%)
3	1 (4%)	5 (5%)	6 (4.8%)
Abortion			
0	20 (80%)	82 (82%)	102 (81.6%)
1	4 (16%)	16 (16%)	20 (16%)
2	1 (4%)	2 (2%)	3 (2.4%)

**Table 2:** Logistic regression model analysis of demographic and hematological parameters of adnexal torsion with respect to ovarian cyst.

	UOR (95% CI)	*p* value	AOR (95% CI)	*p* value
Age	1.000 (0.958–1.043)	0.990	0.958 (0.893–1.027)	0.228
Parity 1	0.779 (0.222–2.736)	0.697	0.791 (0.130–4.793)	0.799
Parity 2	1.270 (0.468–3.441)	0.639	1.974 (0.312–12.487)	0.470
Parity 3	0.818 (0.087–7.731)	0.861	2.565 (0.080–82.324)	0.595
Abortion 1	1.025 (0.309–3.402)	0.968	0.806 (0.110–5.896)	0.832
Abortion 2	2.050 (0.177–23.749)	0.566	1.562 (0.037–66.647)	0.816
WBC	1.000 (1.000–1.001)	0.001	1.000 (1.000–1.000)	0.404
Neutrophil	1.162 (1.095–1.233)	≤0.001	1.075 (0.930–1.241)	0.328
Lymphocyte	0.848 (0.788–0.913)	≤0.001	1.043 (0.902–1.207)	0.567
NLR ratio ≥3	32.019 (10.187–100.636)	≤0.001	26.345 (2.630–263.877)	0.005
Constant		−6.091		

UOR: unadjusted odds ratio; AOR: adjusted odds ratio.

**Table 3:** Hematological parameters of the patients.

Characteristics	Adnexal torsion		Ovarian cyst		Total	
	Median	Q3-Q1	Median	Q3-Q1	Median	Q3-Q1
WBC (per cu mm)	9950	11025–8020	7475	9202.5–5860	8070	9560–5940
Neutrophil	74.4	79.95–63.3	56.7	62.95–51.3	59.4	65.9–51.45
Lymphocyte	21.2	23.4–16	31.15	37.38–26.75	29.7	37.15–23.41
NLR	3.47	4.57–2.9	1.82	2.23–1.4	1.93	2.69–1.41

**Table 4:** Sensitivity and specificity of NLR range (<3 vs. ≥3) in diagnosis of adnexal torsion vs ovarian cyst.

Characteristics	Sensitivity (%)	Specificity (%)	AUC (95% CI)
NLR range (<3 vs. ≥3)	76	91	0.835 (0.745–0.925)

**Table 5:** Operative findings of the patients.

	Adnexal torsion (%)	Ovarian cyst (%)	Total (%)
Operative technique			
Laparoscopy	20 (80%)	87 (87%)	107 (85.6%)
Laparotomy	5 (20%)	13 (13%)	18 (14.4%)
Operative procedure			
Cystectomy	6 (24%)	72 (72%)	78 (62.4%)
Oophorectomy	19 (76%)	28 (28%)	47 (37.6%)
HPE			
Serous tumor	5 (20%)	33 (33%)	38 (30.4%)
Chronic oophoritis	0	1 (1%)	1 (0.8%)
Ovarian fibroma	0	1 (1%)	1 (0.8%)
Mucinous tumor	3 (12%)	14 (14%)	17 (13.6%)
Mature cystic teatime	8 (32%)	13 (13%)	21 (16.8%)
Hemorrhagic luteal cyst	2 (8%)	12 (12%)	14 (11.2%)
Endometriotic cyst	3 (12%)	22 (22%)	25 (20%)
Infarcted ovary	4 (16%)	0	4 (3.2%)
Teratoma + mucinous adenoma	0	1 (1%)	1 (0.8%)
Endometriotic + hemorrhagic	0	1 (1%)	1 (0.8%)
Serous + hemorrhagic	0	2 (2%)	2 (1.6%)

## Data Availability

The data used to support the findings of this study are available from the authors.
